# Excised Abdominoplasty Material as a Systematic Plastic Surgical Training Model

**DOI:** 10.1155/2012/834212

**Published:** 2012-09-26

**Authors:** M. Erol Demirseren, Candemir Ceran, Yakup Duman, Murat Sarici

**Affiliations:** ^1^Department of Plastic, Reconstructive and Aesthetic Surgery, Ataturk Training and Research Hospital, 06810 Ankara, Turkey; ^2^Department of Plastic, Reconstructive and Aesthetic Surgery, Kecioren Training and Research Hospital, 06380 Ankara, Turkey; ^3^Department of Plastic, Reconstructive and Aesthetic Surgery, Bitlis State Hospital, 13000 Bitlis, Turkey

## Abstract

Achieving a level of technical skill and confidence in surgical operations is the main goal of plastic surgical training. Operating rooms were accepted as the practical teaching venues of the traditional apprenticeship model. However, increased patient population, time, and ethical and legal considerations made preoperation room practical work a must for plastic surgical training. There are several plastic surgical teaching models and simulators which are very useful in preoperation room practical training and the evaluation of plastic surgery residents. The full thickness skin with its vascular network excised in abdominoplasty procedures is an easily obtainable real human tissue which could be used as a training model in plastic surgery.

## 1. Introduction 

Academic plastic surgeons have an important role of teaching future plastic surgeons to help them improve their technical skills. Most of this educational practical work is done in operating rooms. However, nowadays this traditional apprenticeship model is slightly shifting to more modern and standardized approaches. Increased patient population, quick turn-over, ethical and legal considerations, and lack of time could be counted as leading factors. Because of these factors supplementary approaches for technical skill training are needed [1, 2]. Abdominoplasty is one of the common procedures performed in plastic surgery departments. The full thickness skin and subcutaneous tissue below the umbilicus are generally excised as a complete island in the procedure. This full thickness skin with its vascular network is a great material to practice surgery on. Keeping this in mind, we aimed to present a practical teaching method which allows the residents to perform basic plastic surgical techniques on human tissue outside the operating room.

## 2. Training Model

**Figure 1:**
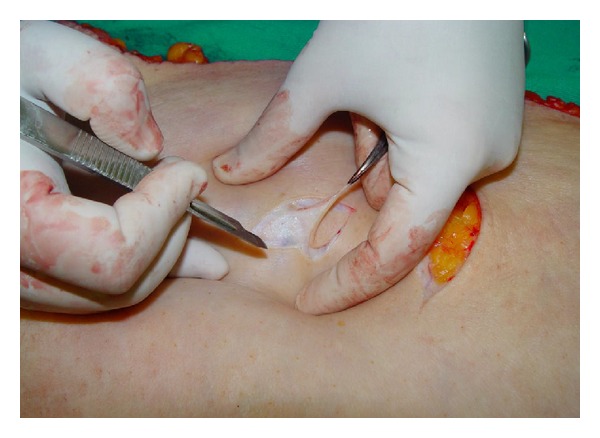
The harvesting of full thickness skin graft by the first-year resident.

**Figure 2:**
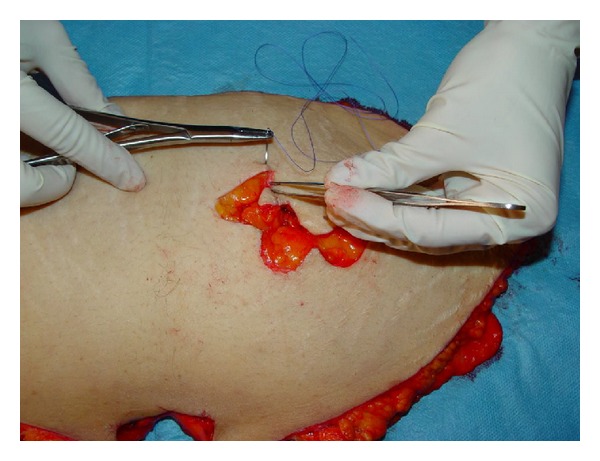
The suturing of the elevated bilobed flap by the second-year resident.

**Figure 3:**
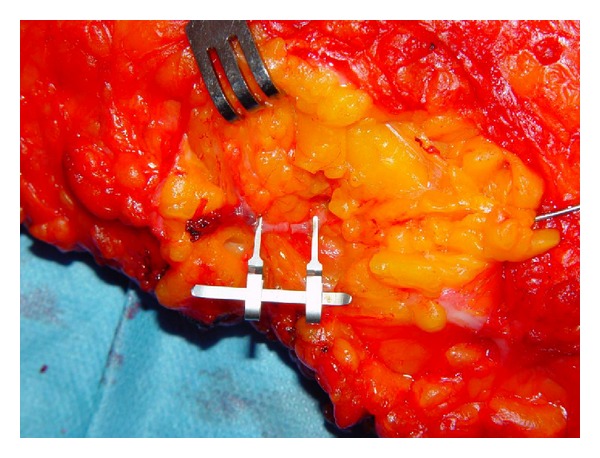
The preparation of end-to-end anastomosis to the artery by the third-year resident.

We designed a practical teaching session for the residents of our department on excised fresh abdominoplasty material. Since abdominoplasty operations are common procedures obtaining material for workshop was not hard. The patient's written approval for this educational session was taken before the planned operation. It was especially planned for the first 3-year residents to have this workshop, but other residents and senior surgeons also participated in this session. Plastic surgical techniques available to study on excised abdominoplasty material were defined as, primary suture technique, full thickness skin graft harvesting, split thickness skin graft harvesting, planning and dissection of local flaps, z-plasty procedures, and microsurgical training according to the resident's level of experience (Table 1). The process was initiated by a short presentation of the procedure by the assigned resident. Following the short presentation, the teaching tenor questioned the resident about the planned procedure. The question process was aimed to determine the resident's level of knowledge about the procedure. The discussion period was always open to all participants. Following the discussion period, the assigned resident was asked to make a plan of the operation on the tissue. If the resident made any mistakes, the senior surgeon corrected the mistakes during the planning stage. The resident performed the operation under the supervision of the senior surgeon on the abdominoplasty material (Figures 1, 2, and 3). The resident performed the operation under supervision of senior surgeon one week after training stage. At the end, all three participating senior surgeons were asked to fill out a questionnaire independently. Answers were counted as in following points; never 1, rarely 2, sometimes 3, often 4, always 5. After the questionnaires have been completed, results were statistically evaluated by using nonparametric test for paired samples as item 1 to item 2 and item 3 to item 4. 

## 3. Results 

Retrospective analysis of the data revealed that 72 excised abdominoplasty materials were used for training between 2004 and 2012. Among the total of 31 residents 11 were first year, 10 were second year, and 10 were third year resident (Table 2). Training sessions performed per year were shown in Table 3. Questionnaire results showed that total duration of procedures was improved and knowledge about the procedures was increased significantly (*P* > 0.05). All participating senior surgeons declared that this training model is helpful (Table 4).

## 4. Discussion

All patients would like to get the best surgical care possible from the most skilled surgeon available. Competence in plastic surgery requires acquisition and proficiency of technical skills which could only be gained by time with repetition and practice of the surgical techniques [3]. Traditionally, plastic surgery residents learn and practice technical skills under the supervision of attending surgeons in the operating room. First, the resident helps the trainer in the operating room and observes the procedures. Then gradually the surgeon in training assumes the role of an operator rather than assistant, and he or she is introduced to the usage of surgical instruments and the principles of dissection, ligation, and suturing under the supervision of a senior surgeon. Eventually, when sufficient skills and confidence have been developed, the residents are allowed to operate on their own under the supervision of the senior surgeon [4]. However, this Halstedian apprenticeship model may no longer be optimal as it is increasingly becoming difficult for the operating room to be the predominant venue for the learning of preoperative planning [5]. 

Pressure on operating room time, the accelerating pace of technical innovation in surgery, greater expectations of the patients, ethical and legal concerns, and the need for surgeons to eliminate the morbidity associated with the learning phase of any new procedure all provide a stimulus for both surgical trainees and trained surgeons to introduce the residents to their teaching techniques before bringing them to the operating room [4]. In addition, economical points are also important since Bridges and Diamond have demonstrated that the cases performed by residents cost significantly more than the cases performed by attending staff alone [6]. 

There are several plastic surgical teaching models and simulators reported in the literature [5, 7–9]. Some of these models are cadaver-or-animal-based models, and the simulators are usually computer-based simulators. We believe that all of these models and simulators are very useful for preoperation room practical training and the evaluation of plastic surgery residents. In our study, we used fresh human tissue excised after abdominoplasty as a surgical training material since it is easy to obtain and supplies real tissue to perform basic plastic surgical techniques. Actually, the use of excised abdominoplasty material for training purposes has been described [10, 11]. But in these models, only microsurgical training and split thickness skin graft harvesting were practiced. In our model, residents performed different plastic surgical techniques according to their level of experience. 

## 5. Conclusions 

We observed that the residents who have basic knowledge could significantly improve their performance on some specific surgical procedures with this preoperative training method. We believe that this will be a step in creating more opportunities for preoperative training models or simulators for the residents.

## Figures and Tables

**Table 1:** Distribution of operations between the residents according to their level.

First year	Second year	Third year
Primary suture technique	Transposition flap	Microdissection of vessels
Full thickness skin graft (FTSG) harvesting	Rotation flap	Adventiciectomy
Split thickness skin graft (STSG) harvesting	V-Y advancement flap	End-to-end anastomosis to artery
	Rhomboid flap	End-to-end anastomosis to vein
	Bilobed flap	End-to-side anastomosis to artery
	Two-flap z plasty	End-to-side anastomosis to vein
	Four-flap z plasty	
	Five-flap z plasty	

**Table 2:** Number of residents participated per year.

Resident level	2004	2005	2006	2007	2008	2009	2010	2011	2012
1	3	1	2		2		2		1
2		3	1	2		2		2	
3			3	1	2		2		2

**Table 3:** Procedures performed per year.

Procedure	Year	2004	2005	2006	2007	2008	2009	2010	2011	2012
	Material	5	6	8	10	9	10	11	8	5
Primary suturing		15	6	16		18		22		5
FTSG harvesting		15	6	16		18		22		5
STSG harvesting		15	6	16		18		22		5
Local flaps			18	8	20		20		16	
z plasty techniques			18	8	20		20		16	
Microsurgical techniques				24	10	18		22		10

**Table 4:** Questions asked to senior surgeons.

No.	Item	Point
1	Does the resident spend the time economically at the training stage?	2.7
2	Does the resident spend the time economically at the operation?	4.1
3	Is the resident's knowledge of procedure sufficient at the training stage?	1.9
4	Is the resident's knowledge of procedure sufficient at the operation?	3.8
5	Do you that this training method is helpful in learning?	5
